# The Regional Centralization of Electronic Fetal Heart Rate Monitoring and Its Impact on Neonatal Acidemia and the Cesarean Birth Rate

**DOI:** 10.1155/2016/3658527

**Published:** 2016-06-09

**Authors:** Kaori Michikata, Hiroshi Sameshima, Hirotoshi Urabe, Syuichi Tokunaga, Yuki Kodama, Tsuyomu Ikenoue

**Affiliations:** Department of Obstetrics and Gynecology, Center for Perinatal Medicine, University of Miyazaki Faculty of Medicine, Miyazaki 889-1692, Japan

## Abstract

*Objective*. The improvement of the accuracy of fetal heart rate (FHR) pattern interpretation to improve perinatal outcomes remains an elusive challenge. We examined the impact of an FHR centralization system on the incidence of neonatal acidemia and cesarean births.* Methods*. We performed a regional, population-based, before-and-after study of 9,139 deliveries over a 3-year period. The chi-squared test was used for the statistical analysis.* Results*. The before-and-after study showed no difference in the rates of acidemia, cesarean births, or perinatal death in the whole population. A subgroup analysis using the 4 hospitals in which an FHR centralization system was continuously connected (compliant group) and 3 hospitals in which the FHR centralization system was connected on demand (noncompliant group) showed that the incidence acidemia was significantly decreased (from 0.47% to 0.11%) without a corresponding increase in the cesarean birth rate due to nonreassuring FHR patterns in the compliant group. Although there was no difference in the incidence of nonreassuring FHR patterns in the noncompliant group, the total cesarean birth rate was significantly higher than that in the compliant group.* Conclusion*. The continuous FHR centralization system, in which specialists help to interpret results and decide clinical actions, was beneficial in reducing the incidence of neonatal acidemia (pH < 7.1) without increasing the cesarean birth rate due to nonreassuring FHR patterns.

## 1. Introduction

Electronic fetal heart rate (FHR) monitoring has been widely used in obstetric practice for more than 4 decades. However, the most recent Cochrane Database systematic review showed that electronic FHR monitoring did not significantly improve the rates of perinatal death (RR 0.86, 95% CI 0.6–1.2, and *n* = 32,000, 11 trials) or cerebral palsy (RR 1.8, 95% CI 0.8–3.6, and *n* = 13,000, 2 trials) in comparison to intermittent auscultation and that it was associated with a significant increase in the rate of cesarean section deliveries (RR 1.6, 95% CI 1.3–2.1, and *n* = 19,000, 11 trials) [1]. Although the international standardization of FHR interpretation has been proposed [2, 3], FHR interpretation is associated with considerable interobserver and intraobserver differences [4]. In addition, there is no consensus in relation to the implementation of FHR pattern-based clinical actions in standardized management [5–7]. To some extent, the ambiguity involved in the interpretation of FHR patterns as well as the clinical actions that are taken based on the interpretation minimizes the scientific value of FHR in clinical settings.

A number of FHR education programs have helped to increase the level of knowledge about FHR interpretation [8–11]. This will hopefully lead to a decrease in the number of poor neonatal outcomes. At the present time, most of the obstetricians in secondary or tertiary centers in Japan have received sufficient training to interpret the most common indeterminate FHR patterns in labor and delivery. In our medical districts, educational programs have been provided with the aim of helping primary obstetricians to reach a consensus on FHR interpretation. However, since the primary obstetricians mainly deal with low-risk pregnancies and rarely experience acidotic newborns, their knowledge is of limited use in interpreting the FHR patterns and determining the appropriate clinical actions in higher risk pregnancies. We hypothesized that if well-educated specialists in the secondary centers are available on a 24-hour basis, then the specialists could help the attending obstetricians in primary hospitals to interpret FHR patterns and provide advice on the appropriate clinical actions at a stage that is early enough to decrease the incidence of neonatal acidemia and intrapartum death. To test this hypothesis, we introduced a centralized FHR network system in one medical district and performed a before-and-after comparison to investigate the incidence of poor perinatal outcomes.

## 2. Methods

This study was approved by the ethics committee of the University of Miyazaki, Faculty of Medicine (numbers 2009-551 and 2014-080).

We performed this study in one medical district in Miyazaki prefecture, which has a population of 280,000. Three thousand deliveries per year were performed in this district from 2011 to 2013. The district includes 2 secondary centers and 7 primary hospitals. The present study included all 7 of the primary hospitals, which mainly dealt with low-risk pregnancies, and 2 secondary centers, which dealt with high-risk pregnancies in women who were referred by the primary hospitals. High-risk factors included preterm labor (≤36 weeks of gestation), multifetal gestation, fetal growth restriction, endocrine disorders, hypertensive disorders, and other obstetric complications. Cesarean deliveries due to intrapartum nonreassuring FHR patterns were usually performed in those primary hospitals. Before the start of this study, all of the 9 hospitals had electronic FHR monitoring devices and the women were continuously monitored after entering the active phase of labor.

In June 2012, we introduced a networking system to centralize the electronic FHR monitoring of the 7 primary hospitals. First, a core networking system was established to connect the 2 secondary centers. Next, each primary hospital was provided with an additional FHR system, which consisted of one central computer and 2 electronic FHR monitoring devices (Atom Medical, Tokyo, Japan). These 7 central computers in the primary hospitals were then connected to the 2-core systems in the secondary centers by a wired networking system. This system enabled the perinatal specialists in the secondary centers to simultaneously evaluate multiple FHR tracings on video displays that were placed in the nursing center, the labor and delivery rooms, and the operating rooms and in other locations. Some of the FHR patterns on the video displays were from the specialists' own patients and others were from patients at the primary hospitals. The scaling used in the FHR display was standardized (the horizontal scaling was 3 cm/min and the vertical scaling was 30 bpm/cm). The FHR interpretation system was applied according to the 1997 guidelines [2].

There were 11 obstetricians in the 7 primary hospitals. The obstetricians were educated on FHR monitoring during their residency; thereafter, they received voluntary reeducation through various clinical conferences. Thus, the individual knowledge levels of the obstetricians about FHR interpretation were variable. They were also able to consult specialists in the secondary center on a 24-hour basis when necessary, even before the start of this study. All 7 of the primary hospitals were able to perform an emergency cesarean section within 30 to 60 minutes during daytime hours.

Five perinatal specialists were accustomed to watching multiple FHR patterns simultaneously on a single video display before the introduction of the network system. In addition to these specialists, some midwives who had been well trained in interpreting FHR patterns were also recruited. After the introduction of the system, at least one specialist watched the FHR patterns on a 24-hour basis. This network system enabled the consultants to watch FHR patterns from the primary hospitals, without knowledge of their labor information. When they noticed abnormal FHR patterns, including recurrent late decelerations, severe variable deceleration, prolonged deceleration, or bradycardia, they called an obstetrician at the attached primary hospital to discuss the interpretation of the FHR patterns and the labor information in order to provide advice on the appropriate clinical actions.

We retrospectively collected data before and after the introduction of the system during two periods. Period 1 was from January 2011 to May 2012. Period 2 was from June 2012 to December 2013. The perinatal data that were collected included the total number of deliveries, the numbers of vaginal deliveries, elective cesarean section deliveries, and emergency cesarean section deliveries due to nonreassuring FHR patterns or other indications, the Apgar scores, and the results of the umbilical arterial blood gas analyses. The details of other indications for emergency cesarean section were not available. We compared the data between periods 1 and 2.

Although this network system was continuously available to all of the 7 primary hospitals, only 4 hospitals used this system continuously (the compliant group). The remaining 3 hospitals submitted FHR patterns only when they felt uncomfortable in interpreting the FHR patterns (the noncompliant group). The percentage of tracings that were actually sent ranged from 0 to 5%. The major difference between the 2 subgroups was that the compliant group received specialist support on a 24-hour basis.

During the last 8 months of period 2, we prospectively collected the contents of the specialist's comments from 773 consecutive intrapartum FHR monitoring sessions. The attending specialists responded to 28 of the 773 FHR tracings from the compliant group. The FHR interpretation and subsequent clinical actions that took place as a result of these 28 FHR tracings were analyzed (Table 4).

We performed a historical cohort study to compare the rates of low pH and cesarean births in the whole study population and then in the compliant and noncompliant groups. The incidences of low pH and cesarean births were compared using the chi-squared test and Fisher's exact test. *P* values of <0.05 were considered to indicate statistical significance.

## 3. Results

We first performed a before-and-after comparison to show the effectiveness of the centralized system in reducing the incidence of acidotic newborns using all of the available data (*n* = 9,139, Table 1). There were no significant differences between the two periods in the prevalence of acidotic infants (pH < 7.1), perinatal deaths in term infants, cesarean births due to nonreassuring FHR patterns, and total cesarean births. We also analyzed the perinatal data of the secondary centers and the primary hospitals separately and found no significant differences between the 2 periods (Table 1).

The network system was connected during labor and delivery for almost all of the cases in the compliant group (>95%). In contrast, the system was connected for <5% of deliveries in the noncompliant group. We therefore performed a subgroup analysis (Table 2). In the compliant group, the incidence of low pH (<7.1) decreased significantly from 4.3/1000 to 1.1/1000 (*P* < 0.03). On the other hand, there was a slight but nonsignificant increase in the incidence of low pH (7.1–7.2). Importantly, the rates of total cesarean births and cesarean births due to nonreassuring FHR patterns did not increase. In the noncompliant group, although there were no significant differences in the rates of low pH (<7.1) or cesarean births due to nonreassuring FHR patterns, there was a significant increase in the total cesarean birth rate (*P* < 0.01). There was no significant difference between the two periods in the incidence of infants with low Apgar scores (Table 2).

The clinical characteristics of 19 cases in which the umbilical blood pH values were <7.1 are shown in Table 3. In the compliant group, umbilical blood pH values of <7.1 were detected in 12 of the 3469 deliveries in period 1. The clinical characteristics of these cases were as follows: terminal bradycardia occurred in 3 cases (Cases 2, 10, and 12), no data was available in 2 cases (Cases 5 and 11), and 1 infant was delivered in an uneventful vaginal delivery (*n* = 1; Case 1). After excluding these unavoidable cases, there were nonreassuring FHR patterns in 6 cases (*n* = 6; 0.17%), who could have benefitted from FHR monitoring. Similarly, the clinical characteristics of the 7 cases of acidosis among the 4,888 deliveries in period 2 were as follows: one case had preexisting hypoxia on admission (Case 14), 2 had an accidental hypoxic event (Cases 15 and 18), and no data was available in 1 case (Case 16). After excluding these unavoidable cases, the remaining 3 showed nonreassuring FHR patterns with acidemia (0.06%, *P* = 0.13 versus 0.17% in period 1). In the compliant group, the corrected incidence (excluding unavoidable cases) of nonreassuring FHR patterns with acidemia was 0.26% (6/2327) in period 1. This frequency decreased to 0.04% (1/2748) in period 2 and showed borderline significance (*P* = 0.053, Fisher's exact test). In the noncompliant group, the corrected incidence of nonreassuring FHR patterns with acidemia did not differ to a statistically significant extent between periods 1 and 2. The specialists were not consulted about all 4 acidotic cases (Cases 16 to 19).

We prospectively collected the contents of the specialist's comments in 773 consecutive intrapartum FHR monitoring tracings over 8 months in period 2. There were a few consultation requests from the noncompliant group regarding the interpretation of intrapartum FHR patterns, but all of them were reassuring. Among the remaining FHR tracings from the compliant group, the specialists generated the call for attention concerning FHR interpretation in 28 tracings (4%) (Table 4). The other 745 tracings were evaluated as reassuring. The most frequent one was the interpretation of late deceleration (originally interpreted as variable deceleration or no deceleration), followed by decreased baseline variability (interpreted as moderate variability). The cases of prolonged deceleration and severe recurrent variable decelerations were interpreted appropriately; however, the attending obstetrician wanted advice regarding clinical actions. The specialists recommended the following actions in these 28 cases: an emergency cesarean section (*n* = 11), expeditious vaginal delivery (*n* = 10), observation (*n* = 6), and a doctor-dispatch (*n* = 1) from the nearby secondary hospital. Only one case (vacuum extraction) resulted in acidosis (Case 13 of Table 3).

## 4. Discussion

We first attempted to show the benefits of FHR centralization in low-risk pregnancies in a single medical district. We found no difference in the incidence of perinatal death, neonatal acidosis (pH < 7.1), or the cesarean birth rate. We then performed a subgroup analysis and showed that when the network system was implemented on a 24-hour basis with compliance, it significantly decreased the incidence of neonatal acidosis without a corresponding increase in the rate of cesarean births (Table 2). With regard to the slight increase in infants with pH 7.1–7.2 in the compliant group, we hypothesize that the specialists' advice was provided early enough to halt the progression of fetuses that were otherwise destined to develop more severe acidosis; however, further investigation should be performed to investigate the reasons for this result.

In the present cohort study, we selected one medical district in which educational programs on FHR interpretation had long been provided for primary obstetricians prior to the start of this study. This is likely to be one of the reasons for the extremely low prevalence of neonatal acidosis (<7.1) in the present study (0.35%, 12/3469 in the primary hospitals) in comparison to our previous study (1.1%) of 5500 unselected low-risk pregnancies [12]. Under these conditions, we performed a before-and-after study and showed that FHR centralization was beneficial in the compliant group. Based on these conditions, we hypothesize that the establishment of FHR centralization in addition to education programs is superior to the provision of education programs alone. Further well-designed studies are required to compare the impacts of the education programs related to FHR interpretation and the FHR centralization system on the perinatal outcomes.

The inter- and intraobserver reliability of FHR interpretation according to the 2008 guidelines have been reported [4]. The intraobserver agreement was almost perfect, while interobserver agreement was poor with regard to baseline FHR variability, especially between absent and minimal FHR variability. In the present study, we did not distinguish absent FHR variability from minimal FHR variability. Instead, we used the term “decreased variability” to describe both absent and minimal FHR variability, because significant FHR deceleration with decreased variability was considered to be sufficient to indicate fetal acidemia [12–15]. Since the interpretation of baseline FHR variability was one of the major points of inquiry in the present study, this FHR centralization system, which enlisted the help of specialists, was considered to be useful in assisting primary obstetricians in making more accurate interpretations.

Centralized multiple monitoring has been reported to be less accurate for detecting critical FHR monitoring signals due to the increased number of displays [16]. As the number of FHR tracings displayed on the screens increased with the addition of the patients from the primary hospitals, the impact of this increase on the interpretation accuracy should be quantitatively studied.

It is important to identify the fetuses that had abnormal FHR patterns from the outset of monitoring and those with a sudden onset of severe hypoxic events, such as cord prolapse and placental abruption because these cases are unlikely to benefit from skilled intervention. Among the acidotic infants in the compliant group, 3 of the 10 infants in period 1 and 2 of the 4 infants in period 2 were found to have had an unavoidable accident (Table 3). After excluding these cases, the incidence of nonreassuring FHR patterns with subsequent neonatal acidemia decreased, with borderline significance, from 0.26% to 0.04% after the introduction of the network system (*P* = 0.053).

The present study is associated with several limitations. One is the possible underestimation of the incidence of abnormal FHR patterns. It was assumed that the specialists would continuously monitor FHR patterns; however, we did not perform a quantitative analysis. Another limitation is that the compliant and noncompliant groups were not randomized; thus some considerable biases existed. For example, there were uncontrollable differences in the capabilities of the clinicians such as their educational background in regard to FHR interpretation. In addition, some differences may have existed in the severity of the cases. Finally, the study population for the FHR investigation was small relative to the low incidence of neonatal acidosis (<1%). Despite these limitations, our regional, population-based study showed that, with the help of specialists to interpret results and determine clinical actions on a 24-hour basis, the centralization of FHR monitoring was useful in achieving a decrease in the rate of fetal acidemia without an increase in the rate of cesarean births.

## Figures and Tables

**Table 1:** The incidence of low pH values, perinatal deaths, and cesarean births before and after the introduction of FHR centralization.

		Period 1 (before)	Period 2 (after)	Statistical significance
All institutions (*n* = 9)	Total deliveries	4251	4888	
	pH values missing	25	10	
	pH < 7.1	14 (0.33%)	14 (0.29%)	ns
	Perinatal deaths at ≥37 w	2	3	ns
	Antepartum deaths	1	2	ns
	Intrapartum deaths	1 (abruption)	0	ns
	Neonatal deaths	0	1 (Potter syndrome)	ns
	CS due to NRFS	260 (6.1%)	265 (5.4%)	ns
	Total CS	1082 (25.5%)	1219 (24.9%)	ns

Secondary hospitals (*n* = 2)	Total deliveries	782	842	
	pH < 7.1	3 (0.38%)	7 (0.83%)	ns
	pH 7.1–7.2	22 (2.8%)	39 (4.6)	ns
	CS due to NRFS	109 (13.9%)	93 (11.0%)	ns
	Total CS	404 (51.7%)	412 (48.9%)	ns

Primary hospitals (*n* = 7)	Total deliveries	3469	4046	
	pH < 7.1	12^*∗*^ (0.35%)	7 (0.17%)	*P* = 0.17
	pH 7.1–7.2	49 (1.4%)	68 (1.7%)	ns
	CS due to NRFS	151 (4.4%)	172 (4.3%)	ns
	Total CS	678 (19.5%)	807 (19.9%)	ns

CS, cesarean section; NRFS, nonreassuring fetal status.

^*∗*^Including 1 intrapartum fetal death of abruption.

**Table 2:** The incidence of low pH values and cesarean births before and after the introduction of FHR centralization in the compliant and noncompliant group.

		Period 1 (before)	Period 2 (after)	Statistical significance
Compliant group (*n* = 4)	Total deliveries	2327	2748	
	CS due to NRFS	89 (3.8%)	93 (3.4%)	ns
	eCS due to reasons other than NRFS	70 (3.0%)	88 (3.2%)	ns
	Total CS	452 (19.4%)	476 (17.3%)	ns
	pH < 7.1	10^*∗*^ (0.43)	3 (0.11%)	*P* = 0.028
	pH 7.1–7.2	43 (1.8%)	58 (2.1%)	ns
	Apgar score < 4 at 1 min	5 (0,2%)	5 (0,2%)	ns
	Apgar score < 4 at 5 min	1 (0.0%)	0	ns

Noncompliant group (*n* = 3)	Total deliveries	1142	1298	
	CS due to NRFS	62 (5.4%)	79 (6.1%)	ns
	eCS due to reasons other than NRFS	20 (1.8%)	31 (2.4%)	ns
	Total CS	226 (19.8%)	331 (25.5%)	*P* = 0.001
	pH < 7.1	2 (0.18%)	4 (0.31%)	ns
	pH 7.1–7.2	6 (0.5%)	10 (0.8%)	ns
	Apgar score < 4 at 1 min	0	2 (0.2%)	ns
	Apgar score < 4 at 5 min	0	0	ns

^*∗*^Including 1 intrapartum fetal death of abruption.

**Table 3:** The clinical characteristics of the 19 acidotic infants.

	Case number	Group	GW (weeks)	Birth weight (g)	Mode of delivery	Apgar score		Umbilical arterial blood gas analysis			Temporal changes in FHR patterns and clinical findings	Associated conditions	Specialist's comments
						1 min	5 min	pH	pCO2	BE			
Period 1	1	Compliant	40	3530	VD	9	9	7.05	59.3	−15.4	Uneventful		
	2	Compliant	39	3320	VD	6	9	7.06	95.8	−7.5	Terminal bradycardia		
	3	Compliant	39	3020	eCS	8	9	7.04	76.5	−12.6	Nonreassuring FHR		
	4	Compliant	41	3170	eCS	6	8	7.02	81.8	−12.5	Nonreassuring FHR		
	5	Compliant	40	3850	VD	5	9	7.05	59	−4.5	Not available		
	6	Compliant	41	3310	eCS	8	8	7.08	60	−13.3	Nonreassuring FHR		
	7	Compliant	39	3210	eCS	3	9	7.07	89.9	−6.9	Nonreassuring FHR		
	8	Compliant	39	2850	eCS	8	9	7.06	76.5	−10.5	Nonreassuring FHR		
	9	Compliant	39	3150	eCS	0	0	—	—	—	Recurrent LD for 5 hours and then bradycardia	Placental abruption	
	10	Compliant	38	2750	VD	8	9	7.09	78	−9.1	Terminal bradycardia		
	11	Noncompliant	38	2570	VD	8	NA	6.83	78.7	−22.7	Not available		
	12	Noncompliant	40	3140	VD	7	8	7.08	56.2	−14.9	Terminal bradycardia		

Period 2	13	Compliant	40	3210	VD	8	9	7.06	79.3	−11.1	Reassuring: rLD for 30 min, followed by terminal PD; vacuum extraction x1		Consultation at 5 min of rLD; vacuum extraction
	14	Compliant	35	2390	VD	3	7	6.95	112	−14.5	Recurrent LD on admission leading to operative vaginal delivery	Abnormal admission test	Preexisting hypoxia Expeditious delivery
	15	Compliant	38	3080	eCS	3	7	6.90	53	−21.4	Sudden onset bradycardia at Cx 6 cm dilatation	Cord prolapse	Unavoidable accident Emergency CS
	16	Noncompliant	37	2730	VD	3	9	6.97	93.6	−12.6	Not available		No consultation
	17	Noncompliant	38	2540	eCS	5	7	7.07	33.6	−20.5	Reassuring: loss of acceleration for 6 hours; occasional LD, and then rLD for 1 hr to decide eCS		No consultation
	18	Noncompliant	39	3180	eCS	3	8	7.00	87.6	−14.2	Reassuring: sudden onset of recurrent severe VDs	Placental abruption	No consultation
	19	Noncompliant	39	3530	VD	7	9	7.09	68.4	−11.5	Reassuring and sudden onset terminal bradycardia (<80 bpm) for 10 min		No consultation

**Table 4:** The fetal heart rate patterns on inquiry during 8 months in period 2 (*n* = 28).

	*n*
(1) Fetal heart rate patterns	
Deceleration	
Late deceleration	22
Prolonged deceleration	9
Variable deceleration	2
Baseline	
Decreased variability	4
Acceleration	2

(2) Recommended clinical actions following abnormal fetal heart rate patterns	
Cesarean section	11
Expeditious vaginal delivery	10
Vaginal delivery	5
Close observation	3
Dispatching doctors	1^*∗*^

^*∗*^This case was also included among the expeditious vaginal delivery cases.
